# Understanding Child Maltreatment and Support Needs in Families with Problematic Parental Substance use 

**DOI:** 10.1177/14550725261422551

**Published:** 2026-02-28

**Authors:** Heidi Rantanen, Ella Plaami, Janika Kosonen, Eija Paavilainen, Katja Kuusisto

**Affiliations:** 1 528748 Health Sciences Unit, Faculty of Social Sciences, Tampere University, Tampere, Finland; 2 528748 Welfare Sciences Unit, Faculty of Social Sciences, Tampere University, Tampere, Finland; 3 The Wellbeing Services County of South Ostrobothnia, Seinäjoki, Finland

**Keywords:** child maltreatment, expert-by-experience, family violence, problematic parental substance use, risk factor, support needs

## Abstract

**Aims:**

This study aims to enhance understanding of child maltreatment (CM) in connection with problematic parental substance use (PPSU) and to identify related individual and familial support needs.

**Methods:**

Using inductive qualitative content analysis, we examined the life stories of trained experts-by-experience (*N* = 11) who had experienced PPSU and various forms of CM, including physical and emotional violence and neglect in their family during childhood and adolescence.

**Results:**

Several family-related and parental risk factors for CM emerged, such as intergenerational transmission of substance use, socioeconomic disadvantage, unemployment or excessive work, parental physical and mental health issues, social wellbeing challenges such as social isolation, violent behaviour, and inadequate parenting practices. Participants also described mitigating factors that partly buffered adverse effects.

**Conclusions:**

The findings highlight diverse and persistent support needs. PPSU can expose children to a wide range of harmful CM experiences. Effective early identification of both PPSU and related CM requires close collaboration between social and health services, as well as inclusive and active client engagement. A holistic understanding of individual and family circumstances affected by PPSU is essential for preventing CM and its intergenerational transmission.

## Introduction

Problematic parental substance use (PPSU) is highly prevalent in Finland, thus demanding efficient services. Approximately 89,000 children have at least one parent with a severe substance use problem (Raitasalo, 2024) and one in 10 Finnish children born in 1991 was affected by PPSU before the age of 18 years (Jääskeläinen et al., 2016b). At the same time, there is a legal demand for parents to hold responsibility for their child's safety and healthy development, whereas social and healthcare professionals are responsible for ensuring that families receive adequate support to meet this responsibility (Child Welfare Act, 417/2007; Government Degree, 338/2011; Health Care Act, 1326/2010). Therefore, harmful conditions in families affected by PPSU create a broad range of service needs. Currently, interventions or integrated programs for children and their families are typically provided by social services, including family-based interventions and support programs targeted at children (Itäpuisto, 2014; Jess et al., 2025), whereas treatment for substance use disorders is typically offered through primary care, mental health services or specialized programs (WHO, 2024).

Child maltreatment (CM) is a chronic and difficult moral problem in societies. CM within the family context encompasses physical, emotional and sexual violence, as well as neglect of children under 18 years, perpetrated by parents or caregivers (WHO, 2016). It also includes living in a violent home environment and witnessing family violence (Chiesa et al., 2018; Holden, 2003; Maughan & Cicchetti, 2002). Violent behavior refers to the intentional use of physical force or power resulting in injury, psychological harm, maldevelopment, deprivation or death (WHO, 2016). CM can also occur unintentionally due to parents’ poor health or lack of knowledge, abilities or resources, failure to act, or carelessness (Rantanen et al., 2022b). CM has long-term consequences for children's physical, psychological and social health (Hughes et al., 2017; WHO, 2016).

CM within the family context is well-studied but remains an extremely complex phenomenon because it is rooted in cultural, economic and social practices (Runyan et al., 2002). One approach is to examine CM through its associated risk factors, which have been shown to increase the likelihood of its occurrence (Milner & Crouch, 2017; Skinner et al., 2021). CM risk factors are widely researched and recognized, although there are some variations between cultures (Rantanen et al., 2022b; WHO 2016). PPSU is one well recognized CM risk factor (Kepple, 2017; Velleman & Templeton, 2007, 2016; Rantanen et al., 2022b). It has been linked to intergenerational transmission and recurrence of CM (Chamberlain et al., 2019; Langevin et al., 2021; White et al., 2015), neglect (Mulder et al., 2018), parent–child attachment problems (Hyysalo et al., 2022), general child well-being issues (Kuppens et al., 2020), obstetric complications and foetal consequences (Shankaran et al., 2007; Sokol, 2003), exposure to family violence (Choenni et al., 2017; Timshel et al., 2017) or intimate partner violence (Cafferky et al., 2018; Chiesa et al., 2018; Choenni et al., 2017; Jernbro et al., 2022), parental mental health problems (Myers et al., 2021), and, in severe cases, homicide (Aho et al., 2017; Stöckl et al., 2017), and medical CM (Yates & Bass, 2017).

PPSU increases the likelihood of physical abuse and neglect, as well as various physical, psychological and cognitive problems in children, such as difficulties with emotion regulation and long-term stress (Freisthler et al., 2021; Punamäki et al., 2021; Raitasalo & Holmila 2017). PPSU often causes feelings of isolation and loneliness in children (Itäpuisto, 2014) and exposes them to multiple social risks, including early problematic substance use, school dropout, poor academic performance, financial insecurity and stigmatization (Jääskeläinen et al., 2016a; Nissinen et al., 2024; O'Shay-Wallace, 2020; Raitasalo et al., 2021). Importantly, Skinner et al. (2021) have highlighted the interconnection of three toxic CM risk factors (i.e. parental substance use, mental health problems and family violence) but cautioned that focusing solely on these in routine practices may divert attention away from other relevant risks. This highlights relevancy to enhance research upon connection between PPSU and various CM risk factors.

Previous research highlights the interconnection between PPSU, CM and the resulting support needs. However, evidence regarding PPSU and CM, its risk factors and support needs remains fragmented, making it insufficient to fully guide multiprofessional social and healthcare practices. Based on this evidence, we aimed at enhancing understanding on the variety of CM risk factors connected to PPSU by (1) exploring how participants describe PPSU connection to CM and its risk factors in their narratives and (2) identifying the types of support needs emerging from these narratives.

## Methods

This study employs a qualitative methodology to explore and describe human experiences in their natural contexts (Kyngäs et al., 2020). PPSU and related CM are not only objective health concerns, but also deeply personal experiences shaped by individual circumstances and cultural contexts. Exploring this diversity enhances understanding of this phenomenon, informs prevention efforts, strengthens client-professional collaboration and supports the development of more effective services for affected individuals and families.

In this study, we drew on informant data from a project that mapped familial risk factors for CM to validate the Family Needs Checklist (Rantanen et al., 2022a). Participants were selected purposefully aiming maximum variation of data meaning a wide range of variation on dimensions of study interest (Polit & Beck 2012). Therefore, we sought to recruit parents of any age and marital status who had diverse experiences related to CM in a family context. The research bulletin listed evidence-based, family-related CM risk factors concerning the parent, child and family context, inviting individuals who had experienced one or more of these factors, including PPSU. Other predetermined inclusion criteria were involvement in support services related to CM, receiving sufficient emotional support to process these experiences, and access to additional support during the study if needed. Participants were recruited through registered associations representing diverse backgrounds. This study ultimately included 11 participants (men and women aged 32–49 years) who were parents with varied marital backgrounds (married, remarried or divorced). All were experts by experience (EbE) with personal histories (McLaughlin, 2009; Pösö, 2018) of PPSU and related maltreatment in childhood and had recovered sufficiently to reflect on their experiences.

In Finland, EbEs are increasingly offered training and monetary compensation for their contributions. Therefore, participants in this study were compensated. The study followed the Finnish National Board on Research Integrity (TENK, 2023) guidelines. Participants provided written informed consent. A checklist (Elo et al., 2014) was used to ensure research quality, and ethical approval was obtained (Ethics Committee of the Tampere Region, 148/2022).

Data were collected through face-to-face, life story interviews in 2023, enabling participants to share personally meaningful memories. Life stories provide rich research material by illuminating key events and contexts related to the phenomenon (Hsieh & Shannon, 2005), thereby deepening the understanding of PPSU and CM. An interview guide was developed with two open-ended questions focusing on childhood CM. At the end of each interview, a structured list of known CM risk factors (Rantanen et al., 2022a) was reviewed to ensure comprehensive coverage of the topic. The guide was piloted with one participant and revised accordingly. Each participant was interviewed once in-person or online enabling nationwide recruitment. Interview duration ranged from 60 to 90 min. All interviews were transcribed verbatim and anonymized due to the sensitivity of the data. During analysis and reporting, confidentiality was secured by anonymizing genders, ages and identifiable life events. Additionally, interviews were pseudonymized to allow traceability during the analysis process without compromising participant confidentiality.

For analysis, we adopted qualitative inductive content analysis (Kyngäs et al., 2020; Polit & Beck, 2012). Data was analysed using ATLAS.ti, version 23.0.8 (https://atlasti.com) and Word (Microsoft Corp., Redmond, WA, USA) by the first investigator (HR). The data were carefully read multiple times. This involved searching for apparent meaningful expressions relevant to the research questions. The data was then organized by assembling and reducing meaningful expressions of how PPSU is connected to CM or its risk, and recognized support needs. Reductions were grouped according to similarities and coded descriptively into subcategories and further into main categories (“PPSU-related CM and associated risk factors” and “support needs”). The findings were checked against the entire dataset. Rigor and accuracy were ensured by comparing transcripts with the derived categories and by presenting the coding process to the research group for review.

## Findings

**Figure 1.**
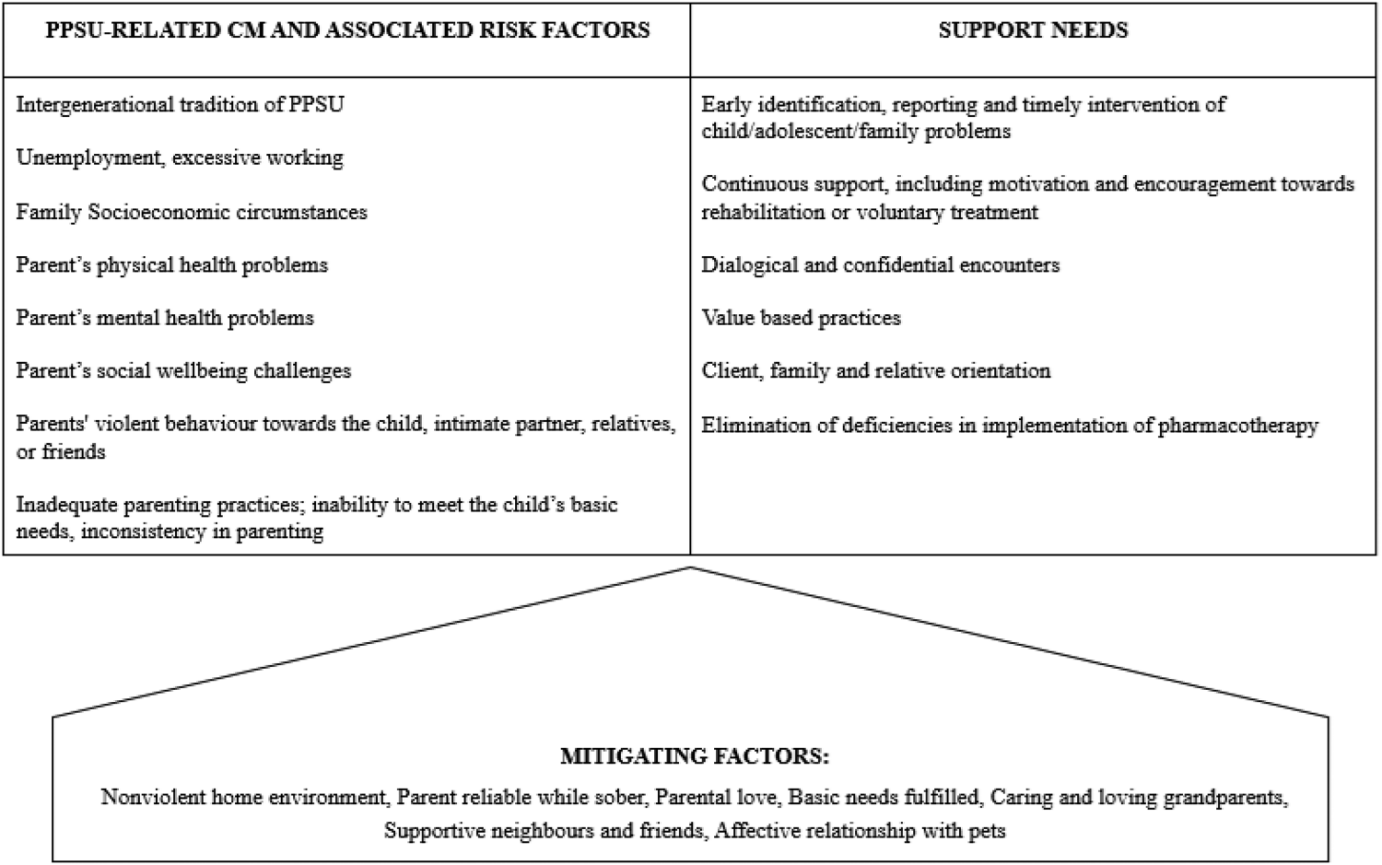
Problematic parental substance use (PPSU)-related child maltreatment (CM), associated risk factors, mitigating factors and support needs identified by participants.

PPSU was experienced as various forms of CM, closely linked to parental and family risk factors, creating unhealthy, uncontrolled and at times unsafe and chaotic living conditions for the child. Participants also described mitigating factors that partially buffered these harmful conditions. In addition, we identified diverse support needs and varied experiences with support services. Figure 1 summarizes the experiences of PPSU-related CM and risk factors, mitigating factors, and identified support needs.

In childhood contexts, PPSU often passed on intergenerationally. Family circumstances varied greatly, reflecting diverse socioeconomic situations and ranging from poverty to wealth, and from periods of parental unemployment to times of excessive working hours: *“At some point in the 90s, the other parent became unemployed* *…* *Then the parent started to make and drink homemade wine at home* *…*” (D4). PPSU was described as ongoing or periodic heavy alcohol use, alcoholism, drug addiction and/or cigarette smoking by one or both parents. Parents openly used alcohol and cigarettes in front of the children, while drug use was more often concealed: *“My parents drank almost every day* *…* *For a long time, I thought it was normal – that every parent drinks every day (laughing)*  *…* *they used drugs when we were sleeping or were not at home*” (D3).

The PPSU habits manifested in varying degrees of physical intoxication, hangovers and fatigue: “*…* *in my family, the hangover got in the way of [hobbies] and [parents] were always pretty tired*” (D10). Sometimes, problematic substance use led to premature death: “*…*  *[parent's death] was kind of the natural end to the lifestyle [parent] had lived* *…*” (D11). Parental mental health problems appeared as work-related stress, depression, mood swings and personality changes: “*When intoxicated, that personality changed. [The parent]* was *always such a warm and trusting person, but when drunk, [the parent] was like a monster*” (D6).

Fluctuations between intoxication and sobriety reflected in parent's social wellbeing and behavior. At home, they could appear emotionally distant and socially isolated, making it difficult or impossible for children to reach out to them. Frequent arguments between parents sometimes led to changes in family structures such as separations, restraining orders, divorce and recurring changes in intimate partners. Non-using parents, by remaining in the relationship, could inadvertently contribute to maintaining harmful circumstances. Daily life in families affected by PPSU was experienced as a constant struggle. Recurrent drinking disrupted routines, and the weekly rhythm revolved around substance use. “*On Mondays [parents] had a hangover, and around Tuesday life became more normal* *…* *On Wednesday it was already ‘nearly weekend’, so [parents] took a little alcohol after sauna. On Thursday [parents] were somewhat apathetic again until Friday, when they were so happy, and the bottles would clink*” (D10). Participants also described a lack of privacy at home, as various people would regularly gather to drink with their parents.

Intoxication, hangovers and fatigue of the parents were experienced as manifesting in parents being short-tempered, violent, unpredictable, distant and at times frightening. Some participants described persistent experiences of violence or fear, which occurred even during periods of parental sobriety. Physical violence or its threat often served a disciplinary purpose or reflected the parent's need to be left alone. “*[The parent] was a devil* *…* *intoxicated all the time* *…* *there wasn’t really any contact* *…*  *I got kicked out if I bothered [the parent] …*  *[The parent] was sometimes sober, too* *…* *but even then, you had to be quiet so [the parent] wouldn’t boil over*” (D11). Children could also be physically harmed when attempting to intervene in violence between adults: “*When my parent's new partner started staying with us more often, the drinking increased, and [the partner] began hitting my parent* *…* *When [the partner] hit me too* *…* *only then did my parent realize the threat. But at first defended and blamed me, saying: ‘There you see, you shouldn’t have come in the middle of our fight’* *…*” (D2).

PPSU manifested as emotional violence through behaviors such as shouting, blaming, invalidation, discrimination, bullying, rejection of physical and emotional closeness, and maintaining cold atmosphere at home: “*In our home, maltreatment was strong discrimination and bullying* *…* *my younger sibling experienced physical violence, but more often it was intense emotional violence directed at us [children] …*” (D7). Participants also witnessed violence between parents, relatives, or their drinking companions: “*Alcohol drinking was associated with sexual violence [between parents]* *…* *I heard screams and rapes at night* *…* *I was a little over ten years old* *…*” (D4).

PPSU was experienced as parents’ inability to meet the child's basic needs, often resulting in neglect of age-appropriate care, attention and nurturing. Parents sometimes compromised children's safety by leaving them alone at night, allowing them to stay out late, or failing to pick them up from kindergarten: “*I was a child* *…* *under school age* *…* *and left completely alone at home. I remember realizing: ‘Aah, I know where they are’,* s*o I left home in the middle of the night and went to the local pub, where I found my parents*” (D8). Basic care routines – such as hygiene, evening structure or nutritious meals – were often neglected: “*…* *Honestly, I only ate proper meals at kindergarten* *…*” (D9). Parents struggled to set boundaries, teach life skills, or provide consistent guidance. To compensate for their substance use, they sometimes resorted to bribing children, reflecting highly inconsistent parenting: “*Alcohol changes the parent's behavior* so much *that, when drunk, [the parent] takes the easiest way out bribing us with money, sometimes setting boundaries, and then ignoring them again*” (D10). Parents also failed to provide social, emotional, or physical closeness. Children were often left alone or sent to play elsewhere, missing shared moments with their parents: “*…* *the parents’ drinking caused us children to spend time all by ourselves* *…*” (D9). When teenagers moved away from home, parents failed to maintain contact, leaving the child unsupported: “*I then moved away from home* *…* *school didn't go well* *…* *I was alone* *…* *I got depressed* *…* *no one cared* *…* *my parents didn't call me* *..*.” (D4).

Participants identified some mitigating factors in their upbringing. Some parents were described as loving, caring, reliable and non-violent, and certain participants felt their basic needs had been met: “*…* *I never thought my parents had put me second, or that alcohol came before me, or that there had been maltreatment* *…* *I always thought I had loving and caring parents*” (D5). Grandparents often provided care, affection, positive attention and leisure activities. Neighbours and friends offered support through practical help, presence and companionship, while pets were seen as important sources of comfort and affection. However, these factors were insufficient to fully mitigate the adverse conditions created by PPSU.

Despite the challenges of PPSU, participants reported several positive experiences of support throughout their lives. External care, such as daycare or out-of-home placements, offered stability and exposure to healthy, typical childrearing. Participants also benefited from a range of supportive services, including professionals specializing in safety and security, family interventions, social welfare, health care, low-threshold crisis support and student welfare. In more severe situations, psychiatric inpatient care was required. Police sometimes intervened to de-escalate violence at home, and social workers provided out-of-home care in cases involving PPSU and family violence affecting young children. Later in life, therapy helped participants make sense of their adverse childhood experiences and supported their recovery.

Participants also reported negative experiences of support, highlighting a lack of comprehensive services that address both family and individual support needs. They particularly emphasized the importance of early identification of PPSU and the need for timely, individualized and family-focused substance use services: “[*In high school], I had anorexia, started using drugs, was self-harming, and had a lot of anxiety, school absences, and skipping classes – but none of these were diagnosed. Personally, I felt depressed and anxious* *…* *and those challenges must have been visible to everyone*” (D1). Participants stressed the importance of continuous support, including encouragement for rehabilitation or voluntary treatment, and the need for confidentiality in care relationships. They also expressed a need to feel valued by professionals and for their views to be heard, understood, and considered in decision-making. In healthcare contexts, some felt like “lost cases” due to the unavailability of treatment facilities and the failure to recognize problematic substance use. They also reported deficiencies in pharmacotherapy, with medications often prescribed based on their own requests – enabling them to rationalize continued use according to the participant's wishes. Some described how their own parents had never received support for addiction or parenting challenges, leading to outdated and inappropriate parenting practices passed across generations. Additionally, participants highlighted a lack of adequate service navigation to help them make use of available support systems.

## Discussion

The present study aimed to enhance understanding of how PPSU is connected to CM, and the types of individual and familial support needs that arise. Several known CM risk factors related to family and parent were identified (Rantanen et al., 2022a; 2022b). Family socioeconomic conditions ranged from poverty to affluence and from unemployment to excessive working hours. Similar patterns have been documented in previous studies (Ronel & Levy-Cahana, 2011; Silvén Hagström & Forinder, 2022). Our findings further demonstrate that PPSU is not exclusively associated with poverty. Moreover, it was reported to persist across generations, underscoring the importance of addressing this cycle in primary prevention strategies targeting PPSU and CM.

Parent-related risk factors encompassed physical, mental, and social dimensions. Physical issues included addiction, intoxication and fatigue; mental health challenges involved depression and stress; and social difficulties reflected strained relationships and non-biological parenthood. Disruptions in parental and stepparent relationships often exposed children to conflict and violence, eroding emotional connection, belonging and social support. PPSU was linked to impaired parenting, including unmet basic needs. From the child's perspective, family life under PPSU involved physical and emotional violence and neglect, even during sobriety. Even seemingly mild maltreatment can feel profound, fostering insecurity from the child perspective. These findings highlight the need to address all forms of child maltreatment comprehensively.

Although mitigating factors were insufficient to fully counteract the adverse conditions described, they nonetheless represent important potential resources. According to Belsky (1993), risk factors do not inevitably lead to poor parenting practices unless stressors outweigh the available supports and override protective factors. Therefore, identifying various forms of CM, associated risks and mitigating factors through dialogue with family members can support a more comprehensive understanding of their unique life situations and guide appropriate care and support. For example, Wlodarczyk et al. (2017), in a systematic review, identified protective mental health factors at the level of the child, parent, family and environment – one of the most significant being social support, which was also reflected in our study. Protective factors against CM constitute a critical component of prevention strategies (WHO, 2016). Further research is needed to identify mitigative and protective factors associated with PPSU.

Participants emphasized the importance of early identification, reporting, and timely intervention for child and family problems. PPSU is recognized as a critical screening criterion for CM (Kepple, 2017). Prior research indicates that high alcohol tolerance and frequent adolescent intoxication increase the risk of problematic substance use (Sarala et al., 2020), whereas protective factors include time spent with parents and structured leisure activities (Woodward et al., 2023). Direct engagement with young people is therefore essential (Stone et al., 2023). Although family violence is closely associated with PPSU, it often remains undetected in social and healthcare settings (Leppäkoski et al., 2024), underscoring the need for active and effective screening. Furthermore, adverse outcomes have been observed even among children whose parents do not meet clinical criteria for substance dependence, highlighting the importance of early intervention (McGovern et al., 2020).

Effective intervention for child and family problems requires a multifaceted approach that integrates early identification and timely action, continuous support, dialogical and confidential encounters, value-based practices, family-oriented care, and the elimination of pharmacotherapy deficiencies. A study by Barret et al. (2024) similarly emphasized the need for timely, individualized and family-focused substance use treatment. However, when caregivers sought help for themselves, services often prioritized safeguarding children, leaving the caregiver without adequate support or ending it prematurely (Barret et al., 2024). This highlights the importance of comprehensive, client- and family-oriented support based on multidisciplinary collaboration. The importance of both individual and family-focused support is emphasized (Kedzior et al., 2024). McGovern et al. (2021) found evidence that tailored psychosocial interventions help sustain reduced PPSU in the long term. Involving other family members in treatment was found to be beneficial, while including children in sessions was not. Parent skills training implemented at home was shown to be effective. These findings underline the need to support both parenting and substance use recovery simultaneously. In their review, Straussner and Fewell (2018) identified several promising interventions for children living with PPSU, including substance use treatment (Burdzovic Andreas & O’Farrell, 2017), family-based programs (Calhoun et al., 2015; Ryan et al., 2016; Usher et al., 2015; Usher & McShane, 2016; Zehetner et al., 2017; Zhang & Slesnick, 2017, 2018), brief interventions (Kepple, 2017; Keurhorst et al., 2016) and out-of-home care prevention models (Huebner et al., 2021).

Our study emphasized the importance of value-based practices, where clients feel respected by professionals and experience being heard, understood and included in decision-making. Confidentiality was also seen essential for discussing sensitive issues without fear of stigma. Early dialogue with families is regarded as valuable addressing concerns (Eriksson & Arnkil, 2009; Seikkula & Arnkil, 2014). It is essential that all individuals in society have access to information about PPSU and its connection to CM, as well as related risk factors, to recognize potential harms and seek early support. Similarly, professionals across sectors must be equipped to understand PPSU as a phenomenon that requires multidisciplinary client- and family-oriented collaboration. Active client participation enhances communication and fosters inclusive care practices.

## Strengths and Limitations

Life story interviews offer opportunities for rich insights into complex phenomena. In this study, interviews originally focused on broader experiences of CM, with PPSU emerging as one of several recognized risk factors. Among those interviews, some informants were selected based on their experiences with PPSU, identified as one of their most harmful childhood experiences. The aim was not to establish causal links between PPSU and CM, but rather to explore and describe the multifaceted realities of participants’ lives. Accordingly, the findings provide valuable insights to support the enhancement of multidisciplinary care and service planning. The limitations of this study relate to potential biases, including participant motivation and memory bias (Baldwin et al., 2019). Participants may have withheld information about CM due to unpleasant emotions, shame, reluctance to revisit harmful events, or fear of disclosure to authorities. Memory bias is an acknowledged limitation in retrospective studies because under- and over-reporting cannot be fully avoided (Baldwin et al., 2019). In this study, trained EbEs served as informants, aiming to minimize this bias. In adulthood, after receiving adequate emotional support to process experiences of CM, many participants were able to recognize forms of CM that had gone unidentified during childhood. The possibility of over-reporting of subjects most important to the participant is inevitably present. Using interviews guided by a structured protocol enabled a detailed and well-grounded exploration of the concept of CM (Baldvin et al., 2019), while also ensuring comprehensiveness and addressing potential gaps or limitations in participants’ understanding.

Data saturation regarding CM risk factors was achieved by systematically addressing them at the end of each interview (Kyngäs et al., 2020), although saturation on PPSU specifically may have remained incomplete due to the broad scope of the CM construct. The goal of this study was merely to enhance understanding of human experience than to achieve generalizability in target population (Polit & Beck, 2012). Single human experiences are unique and thus cannot be fully generalized. They describe the existing reality within a human experience and hold intrinsic value. Additionally, subjective client perspectives and participation in service planning are mandatory part of inclusive service development.

## Practical Implications

From a policy perspective, priority should be given to promoting child and family welfare and reducing PPSU, rather than prioritizing the financial interests of substance policies. PPSU must be recognized as a serious societal issue that demands urgent and coordinated action. Community must work toward developing practices that shift from viewing cases as lost or irretrievable to recognizing them as hopeful and recoverable, and from stigmatization to embracing the complexity of human life. Social and healthcare professionals are encouraged to: (1) strengthen the early identification of PPSU, associated CM, and related risk factors; (2) detect child and adolescent difficulties at an early stage; and (3) implement and further develop effective, client- and family-centered interventions in collaboration with relevant service providers. Policymakers are advised to: (1) acknowledge PPSU as a serious health and well-being concern for both children and parents and (2) apply existing robust evidence on PPSU connections to CM when making substance related policy decisions affecting wellbeing of families with children.

## Conclusions

Individual experiences of PPSU vividly illustrate the lived experiences of children and highlight the urgent need for early preventive actions. These findings offer valuable insights into the harmful conditions within PPSU-affected families, which may span generations and result in unsafe family environments from the child's perspective. Notably, PPSU was often perceived as a normal part of everyday life, extending even to parents’ sober moments. Despite the presence of mitigating factors, its effects on participants’ health and well-being were severe and far-reaching. Importantly, children do not always perceive PPSU as negative in the moment. Realization of harm may only emerge later; for example, through therapeutic work in adulthood. From a sustainable development perspective, the key priority is the early identification of individual and familial challenges and the timely provision of multidisciplinary support. Recognizing the diversity of risk factors and sharing knowledge across sectors benefits children, parents, professionals and systems alike by fostering a shared understanding of complex support needs. Effective care must be built on joint governance in multiprofessional work, where the client's unique life situation serves as the starting point for planning, prioritizing and delivering support. Care processes should be shaped by the client's perspective, ensuring that structures of support are responsive, timely and meaningful.
